# Supplementary material for: Perceptions and experiences of the prevention, testing, and treatment of anaemia in pregnant women: A qualitative evidence synthesis
Source: PLOS Glob Public Health. 2025 Oct 1;5(10):e0005158. doi: 10.1371/journal.pgph.0005158 (PMC12488017; doi:10.1371/journal.pgph.0005158)
Supplement: S2 Appendix — (DOCX) [file pgph.0005158.s002.docx]

**S2 Appendix: Search strategies**

**Medline**

| **#** | **Searches** |
| --- | --- |
| **1** | anemi*.mp. |
| **2** | anaemi*.mp. |
| **3** | iron.mp. |
| **4** | hemoglobin.mp. |
| **5** | haemoglobin.mp. |
| **6** | hematocrit.mp. |
| **7** | haematocrit.mp. |
| **8** | 1 or 2 or 3 or 4 or 5 or 6 or 7 |
| **9** | exp anemia/ |
| **10** | 8 or 9 |
| **11** | pregnan*.mp. |
| **12** | gestation*.mp. |
| **13** | obstetri*.mp. |
| **14** | prenatal.mp. |
| **15** | antenatal.mp. |
| **16** | 11 or 12 or 13 or 14 or 15 |
| **17** | 10 and 16 |
| **18** | limit 17 to “qualitative (best balance of sensitivity and specificity)” |
| **19** | qualitative research/ |
| **20** | 17 and 19 |
| **21** | 18 or 20 |

**CINAHL**

| **#** | **Searches:** |
| --- | --- |
| **S1** | anemi* |
| **S2** | anaemi* |
| **S3** | iron |
| **S4** | hemoglobin |
| **S5** | haemoglobin |
| **S6** | hematocrit |
| **S7** | haematocrit |
| **S8** | S1 or S2 or S3 or S4 or S5 or S6 or S7 |
| **S9** | (MH “Anemia+”) |
| **S10** | S8 or S9 |
| **S11** | pregnan* |
| **S12** | gestation* |
| **S13** | obstetri* |
| **S14** | prenatal |
| **S15** | antenatal |
| **S16** | S11 or S12 or S13 or S14 or S15 |
| **S17** | S10 and S16 |
| **S18** | S17 Limiters – Clinical Queries: Qualitative – Best Balance |
| **S19** | (MH “Qualitative Studies+”) |
| **S20** | S17 and S19 |
| **S21** | S18 or S20 |

**Scopus**

 ( TITLE-ABS KEY ( anemi*  OR  anaemi*  OR  iron  OR  hemoglobin  OR  haemoglobin  OR  hematocrit  OR  haematocrit ) )  AND  ( TITLE-ABS-KEY ( pregnan*  OR  gestation*  OR  obstetri*  OR  prenatal  OR  antenatal ) )  AND  ( ( KEY ( "qualitative research"  OR  interview  OR  "semi-structured interview"  OR  "thematic analysis"  OR  "qualitative analysis" ) )  OR  ( TITLE-ABS ( qualitative  OR  interview*  OR  "thematic analysis"  OR  themes  OR  "mixed method"  OR  "mixed methods" ) ) )
