# Supplementary material for: Perceptions and experiences of the prevention, testing, and treatment of anaemia in pregnant women: A qualitative evidence synthesis
Source: PLOS Glob Public Health. 2025 Oct 1;5(10):e0005158. doi: 10.1371/journal.pgph.0005158 (PMC12488017; doi:10.1371/journal.pgph.0005158)
Supplement: S3 Appendix — (DOCX) [file pgph.0005158.s003.docx]

**S3 Appendix: GRADE-CERQual evidence profile**

| **Findings** | **Summary of qualitative review findings** | **Contributing qualitative studies** | **Methodological limitations** | **Coherence** | **Relevance** | **Adequacy** | **Overall CERQual assessment** | **Explanation of overall assessment** |
| --- | --- | --- | --- | --- | --- | --- | --- | --- |
|  | **1.0 Socio-cultural context of anaemia in pregnant women** | | | | | | | |
| 1.1 | **Women and community members’ knowledge of anaemia in pregnant women.** Most women and community members were not aware of the term ‘anaemia’, and often referred to anaemia as ‘lack of blood’ or described it in terms of symptoms (e.g., fatigue and dizziness). Some women and community members had knowledge of more severe, long-term consequences of anaemia in pregnant women. Anaemia in pregnancy was often attributed to the woman sharing her blood with the fetus and an inadequate intake of nutritious food. | [34, 36, 44-47, 51, 56-59, 63, 66, 72-75, 78, 81, 85, 87-91, 93, 94] | Minor concerns: 3 papers with no or very minor concerns, 12 papers with minor concerns (recruitment, reflexivity, ethics, data analysis), 8 papers with moderate concerns (recruitment, reflexivity, data analysis), and 4 papers with serious concerns (recruitment, reflexivity, ethics, data collection, data analysis). | No or very minor concerns | No or very minor concerns: 24 papers directly and 3 papers indirectly relevant to review aim. Contributing papers represented 5 regions, Africa (Ethiopia, Burkina Faso, Nigeria, Malawi, Kenya, South Africa, Zimbabwe, Ghana, Uganda, Tanzania), South-East Asia (Bangladesh, India, Indonesia, Cambodia, Nepal), Americas (Bolivia, Guatemala, Honduras, Argentina), Eastern Mediterranean (Pakistan), and Europe (Ireland), 21 countries where 1 is a high-income country, 2 are upper-middle income countries, 10 are lower-middle income countries, and 8 are low-income countries. Perspectives came from pregnant women, non-pregnant women, partner/family members, health workers, lay health workers, and cultural/religious/community members. | No or very minor concerns: 27 out of 61 papers contributed to the review finding (20 papers with moderate to thick data and 7 papers with thin data). | High confidence | No or very minor concerns about coherence, adequacy, and relevance; minor concerns about methodological limitations (recruitment, reflexivity, ethics, data collection, and data analysis). |
| 1.2 | **Women and community members’ beliefs about anaemia in pregnant women.** Many women and community members considered common symptoms of anaemia (e.g., fatigue, weakness, and dizziness) as ‘normal during pregnancy’ and therefore not concerning. These beliefs were often passed down from older family members or from seeing other women in the community endure these symptoms during pregnancy. However, women sought care from health workers if anaemia symptoms hindered their ability to work, if they believed they would have a difficult childbirth, or if symptoms were perceived as affecting the fetus. | [35, 50, 51, 54, 56, 57, 59, 75, 78, 79, 88, 90, 93, 94] | Moderate concerns: 2 papers with no or very minor concerns, 5 papers with minor concerns (recruitment, reflexivity, data analysis), 2 papers with moderate concerns (recruitment, reflexivity, data analysis), and 5 papers with serious concerns (qualitative methodology, research design, recruitment, reflexivity, ethics, data collection, data analysis). | No or very minor concerns | Minor concerns: All 14 papers directly relevant to the review aim. Contributing papers represented 4 regions, Africa (Nigeria, Malawi, Burkina Faso, Kenya), Americas (Bolivia, Guatemala, Honduras, Argentina), South-East Asia (India, Indonesia, Thailand), and Eastern Mediterranean (Pakistan), 12 countries where 2 are upper-middle income countries, 8 are lower-middle income countries, and 2 are low-income countries. Perspectives came from pregnant women, non-pregnant women, partner/family members, health workers, lay health workers, and traditional birth attendants. | Minor concerns: 14 out of 61 papers contributed to the review finding (10 papers with moderate to thick data and 4 papers with thin data). | Moderate confidence | No or very minor concerns about adherence, minor concerns about adequacy (14 papers; 10 with moderate to thick data richness and 4 with thin data richness), minor concerns about relevance (4 regions; 2 upper-MICs, 8 lower-MICs, and 2 LICs), and moderate concerns about methodological limitations (qualitative methodology, research design, recruitment, reflexivity, ethics, data collection, data analysis). |
| 1.3 | **Positive perceptions about taking traditional medicine to manage anaemia in pregnancy.** Women, health workers, lay health workers, and community members described several reasons why women may take traditional medicines to prevent and/or treat anaemia in pregnancy. Reasons included where anaemia was attributed to black magic or a curse, beliefs that traditional medicine was more effective than Western medicine, challenges accessing antenatal care services, preference to not take medicine (i.e., pills) during pregnancy, and women having experienced side-effects with supplements. | [34, 37, 39, 43, 45, 47, 53, 60, 78, 82, 91, 93] | Moderate concerns: 2 papers with no or very minor concerns, 4 papers with minor concerns (recruitment, reflexivity), 4 papers with moderate concerns (recruitment, reflexivity, data collection, data analysis), and 2 papers with serious concerns (reflexivity, ethics, data analysis, recruitment). | No or very minor concerns | Minor concerns: 11 papers directly and 1 paper indirectly relevant to review aim. Contributing papers represented 4 regions, Africa (Malawi, Burkina Faso, Zimbabwe, Nigeria, Kenya, Ghana, Uganda, South Africa, Tanzania), Americas (Bolivia, Guatemala, Honduras), South-East Asia (India, Indonesia), and Eastern Mediterranean (Pakistan), 15 countries where 1 is an upper-middle country, 7 are lower-middle income countries, and 7 are low-income countries. Perspectives came from pregnant women, non-pregnant women, partner/family members, health workers, lay health workers, traditional birth attendants, and cultural/religious/community leaders. | Moderate concerns: 12 out of 61 papers contributed to the review finding (3 papers with moderate to thick data and 9 papers with thin data). | Low confidence | No or very minor concerns about coherence, minor concerns about relevance (4 regions; 1 upper-MIC, 7 lower-MICs, and 7 LICs), moderate concerns about adequacy (12 papers; 3 with moderate to thick data richness and 9 with thin data richness), and moderate concerns about methodological limitations (recruitment, reflexivity, ethics, data collection, and data analysis). |
| 1.4 | **Fears about traditional medicine to manage anaemia in pregnancy.** Some women were pressured by family members (e.g., mothers-in-law) to take traditional medicine to prevent and/or treat anaemia in pregnancy. Women feared that if they did not take traditional medicine, it would be perceived as insubordination to family authority and they would be blamed for any complications during childbirth (e.g., caesarean section). Conversely, some women were concerned that taking traditional medicine may be harmful to the fetus and cause a miscarriage. | [53, 75, 91, 94] | Moderate concerns: 2 papers with minor concerns (reflexivity) and 2 papers with serious concerns (recruitment, reflexivity, ethics, data collection, data analysis). | No or very minor concerns | Serious concerns: All 4 papers directly relevant to review aim. Contributing papers represented 1 region, Africa (Nigeria, Zimbabwe, Kenya, Tanzania), 4 countries where 2 are lower-middle income countries and 2 are low-income countries. Perspectives came from pregnant women, non-pregnant women, health workers, lay health workers, and traditional birth attendants. | Serious concerns: 4 out of 61 papers contributed to the review finding (1 paper with thick data and 3 papers with thin data). | Very low confidence | No or very minor concerns about coherence, moderate concerns about methodological limitations (recruitment, reflexivity, ethics, data collection, and data analysis), serious concerns about adequacy (4 papers; 1 with thick data richness and 3 with thin data richness), and serious concerns about relevance (1 region; 2 lower-MICs and 2 LICs). |
| 1.5 | **Limited decision-making power and social position of women affects their management of anaemia in pregnancy.** Many women and some health workers and lay health workers reported that women were often not involved in decision-making around supplementation and diet during their pregnancy, with decisions made by the woman’s family members (commonly their mother-in-law or husband). The heavy workload of women and social norms around prioritising family wellbeing over their own also limited women’s ability to access anaemia management care. | [42, 43, 45, 46, 52, 53, 56, 59, 81, 82, 85, 86, 88, 93] | Moderate concerns: 1 paper with no or very minor concerns, 7 papers with minor concerns (recruitment, reflexivity, ethics, data analysis), 5 papers with moderate concerns (recruitment, reflexivity, data analysis), and 1 paper with serious concerns (reflexivity, ethics, data analysis). | No or very minor concerns | Minor concerns: 13 papers directly and 1 paper indirectly relevant to review aim. Contributing papers represented 4 regions, South-East Asia (Bangladesh, India, Indonesia, Nepal), Africa (Burkina Faso, Malawi, Zimbabwe, Nigeria), Americas (Bolivia, Guatemala, Honduras), and Eastern Mediterranean (Pakistan), 12 countries where 8 are lower-middle income countries and 4 are low-income countries. Perspectives came from pregnant women, non-pregnant women, partner/family members, health workers, lay health workers, traditional birth attendants, cultural/religious/community leaders, and non-governmental organisation staff. | Minor concerns: 14 out of 61 papers contributed to the review finding (8 papers with moderate to thick data and 6 papers with thin data). | Moderate confidence | No or very minor concerns about coherence, minor concerns about adequacy (14 papers; 8 with moderate to thick data richness and 6 with thin data richness), minor concerns about relevance (4 regions; 8 lower-MICs and 4 LICs), and moderate concerns about methodological limitations (recruitment, reflexivity, ethics, and data analysis). |
| 1.6 | **Delayed or inconsistent antenatal care affects the management of anaemia in pregnant women.** Many women, community members, health workers and lay health workers described how these delays affected the management of anaemia in pregnant women, such as insufficient time for women to take the recommended number of supplements. Women delayed or attended antenatal care inconsistently for various reasons, including a reluctance to disclose pregnancy due to fear of community gossip and vulnerability to evil spirits, limited healthcare decision-making power, distance to health facilities, long waiting times at health facilities, poor attitudes of health workers, the perception that care was unnecessary due to the absence of complications, and associated costs (e.g., transport). | [34, 52, 55-58, 62, 65, 69, 74, 75, 78, 85, 86, 89, 93] | Moderate concerns: 1 paper with no or very minor concerns, 8 papers with minor concerns (reflexivity, data analysis), 6 papers with moderate concerns (recruitment, reflexivity, data analysis), and 1 paper with serious concerns (recruitment, ethics, data analysis). | No or very minor concerns | Minor concerns: 15 papers directly and 1 paper indirectly relevant to review aim. Contributing papers represented 4 regions, Africa (Kenya, Burkina Faso, Malawi, Tanzania, Nigeria, Ethiopia, Zimbabwe, Ghana, Uganda), South-East Asia (Bangladesh, Indonesia, India, Cambodia, Nepal), Americas (Bolivia, Guatemala, Honduras), and Eastern Mediterranean (Pakistan), 18 countries where 1 is an upper-middle income country, 10 are lower-middle income countries, and 7 are low-income countries. Perspectives came from pregnant women, non-pregnant women, partner/family members, health workers, lay health workers, traditional birth attendants, cultural/religious/community leaders, and non-governmental organisation staff. | Minor concerns: 16 out of 61 papers contributed to the review finding (10 papers with moderate to thick data and 6 papers with thin data). | Moderate confidence | No or very minor concerns about coherence, minor concerns about adequacy (16 papers; 10 with moderate to thick data richness and 6 with thin data richness), minor concerns about relevance (4 regions; 1 upper-MIC, 10 lower-MICs, and 7 LICs), and moderate concerns about methodological limitations (recruitment, reflexivity, ethics, and data analysis). |
|  | **2.0 Prevention and/or treatment of anaemia in pregnant women through diet, supplementation, or clinical intervention** | | | | | | | |
| 2.1 | **Importance of a nutritious diet to manage anaemia in pregnancy.** Many health workers, lay health workers, women, and community members described that a nutritious diet was essential for preventing and/or treating anaemia in pregnant women, as they shared their food and blood with the fetus. Some women’s diets changed during pregnancy to include more vegetables and fruits, larger portions, and to eat first rather than last within their families. Some women expressed a preference for managing anaemia through diet rather than supplementation, viewing food as natural and containing all necessary iron. | [34, 36, 43, 44, 46, 52, 56-58, 66, 70, 73-75, 78, 79, 81, 85, 87, 88, 90, 91, 93, 94] | Moderate concerns: 1 paper with no or very minor concerns, 10 papers with minor concerns (recruitment, reflexivity, ethics, data analysis), 8 papers with moderate concerns (recruitment, reflexivity, data analysis), and 5 papers with serious concerns (recruitment, reflexivity, ethics, data collection, data analysis). | No or very minor concerns | No or very minor concerns: 22 papers directly and 2 papers indirectly relevant to review aim. Contributing papers represented 5 regions, Africa (Ethiopia, Burkina Faso, Nigeria, Malawi, Kenya, Zimbabwe, Ghana, Uganda, Tanzania), South-East Asia (Bangladesh, India, Indonesia, Nepal), Americas (Bolivia, Guatemala, Honduras, Argentina), Eastern Mediterranean (Pakistan), and Europe (Ireland), 20 countries where 1 is a high-income country, 2 are upper-middle income countries, 10 are lower-middle income countries, and 7 are low-income countries. Perspectives came from pregnant women, non-pregnant women, partner/family members, health workers, lay health workers, traditional birth attendants, cultural/religious/community leaders, and non-governmental organisation staff. | Minor concerns: 24 out of 61 papers contributed to the review finding (12 papers with moderate to thick data and 12 papers with thin data). | Moderate confidence | No or very minor concerns about coherence and relevance, minor concerns about adequacy (24 papers; 12 with moderate to thick data richness and 12 with thin data richness), and moderate concerns about methodological limitations (recruitment, reflexivity, ethics, data collection, and data analysis). |
| 2.2 | **Challenges to women having a nutritious diet to manage anaemia in pregnancy.** Many women and some community members, health workers, and lay health workers described how the high cost of nutritious food was a barrier to women having a nutrient-rich diet to prevent and/or treat anaemia in pregnancy. Additional barriers to women having a nutritious diet in pregnancy included limited decision-making power and social positionality of women, low availability of nutritious foods in some settings, inadequate knowledge of iron-rich foods, consumption of food and drink that inhibited iron absorption, and food taboos and religious beliefs that prevented pregnant woman from eating certain foods (e.g., animal products). | [35, 42, 44, 46, 50-54, 56, 57, 65, 70, 73-75, 77, 81, 82, 85, 88, 93, 94] | Moderate concerns: 1 paper with no or very minor concerns, 12 papers with minor concerns (recruitment, reflexivity, ethics, data analysis), 7 papers with moderate concerns (recruitment, reflexivity, data collection, data analysis), and 3 papers with serious concerns (recruitment, reflexivity, ethics, data analysis). | No or very minor concerns | No or very minor concerns: 22 papers directly and 1 paper indirectly relevant to review aim. Contributing papers represented 4 regions, Africa (Ethiopia, Nigeria, Burkina Faso, Malawi, Zimbabwe, Kenya), South-East Asia (Bangladesh, India, Indonesia, Thailand, Nepal), Americas (Bolivia, Guatemala, Honduras), and Eastern Mediterranean (Pakistan), 15 countries where 1 is an upper-middle income country, 10 are lower-middle income countries, and 4 are low-income countries. Perspectives came from pregnant women, non-pregnant women, partner/family members, health workers, lay health workers, traditional birth attendants, cultural/religious/community leaders, and non-governmental organisation staff. | Minor concerns: 23 out of 61 papers contributed to the review finding (16 papers with moderate to thick data and 7 papers with thin data). | Moderate confidence | No or very minor concerns about coherence and relevance, minor concerns about adequacy (23 papers; 16 with moderate to thick data richness and 7 with thin data richness), and moderate concerns about methodological limitations (recruitment, reflexivity, ethics, data collection, and data analysis). |
| 2.3 | **Characteristics of supplements.** Some women discussed the bitter taste, smell, and large size of supplements as negatively affecting adherence. | [35, 38, 39, 42, 43, 47, 52, 59, 60, 63, 75] | Minor concerns: 2 papers with no or very minor concerns, 3 papers with minor concerns (recruitment, reflexivity), and 5 papers with moderate concerns (recruitment, reflexivity, data collection, data analysis). | No or very minor concerns | Moderate concerns: 10 papers directly and 1 paper indirectly relevant to review aim. Contributing papers represented 2 regions, Africa (Kenya, South Africa) and South-East Asia (India, Nepal, Indonesia), 5 countries where 2 are upper-middle income countries and 3 are lower-middle income countries. Perspectives came from pregnant women, non-pregnant women, health workers, lay health workers, traditional birth attendants, cultural/religious/community leaders, and non-governmental organisation staff. | Serious concerns: 11 out of 61 papers contributed to the review finding (1 paper with moderate to thick data and 10 papers with thin data). | Low confidence | No or very minor concerns about coherence, minor concerns about methodological limitations (recruitment, reflexivity, data collection, and data analysis), serious concerns about adequacy (11 papers; 1 with moderate to thick data richness and 10 with thin data richness), and moderate concerns about relevance (2 regions; 2 upper-MIC and 3 lower-MICs). |
| 2.4 | **Side-effects of supplements.** Many women reported not adhering to supplements after experiencing side-effects, including nausea, dizziness, vomiting, diarrhoea, constipation, blackened stool, heartburn, loss or increase of appetite, and/or indigestion. | [34-39, 41-43, 45-47, 52, 53, 57-60, 62, 63, 72-75, 84-86, 88, 93] | Moderate concerns: 3 papers with no or very minor concerns, 13 papers with minor concerns (recruitment, reflexivity, ethics), 12 papers with moderate concerns (recruitment, reflexivity, data collection, data analysis), and 1 paper with serious concerns (reflexivity, ethics, data analysis). | No or very minor concerns | No or very minor concerns: 26 papers directly and 3 papers indirectly relevant to review aim. Contributing papers represented 5 regions, Africa (Ethiopia, Burkina Faso, Malawi, Tanzania, Kenya, South Africa, Nigeria, Ghana, Uganda, Zimbabwe), South-East Asia (Bangladesh, India, Indonesia, Nepal), Americas (Bolivia, Guatemala, Honduras), Eastern Mediterranean (Pakistan), and Europe (Ireland). 19 countries where 1 is a high-income country, 1 is an upper-middle income country, 12 are lower-middle income countries, and 5 are low-income countries. Perspectives came from pregnant women, non-pregnant women, partner/family members, health workers, lay health workers, and cultural/religious/community leaders. | Moderate concerns: 29 out of 61 papers contributed to the review finding (6 papers with moderate to thick data and 23 papers with thin data). | Moderate confidence | No or very minor concerns about coherence and relevance, moderate concerns about adequacy (29 papers; 6 with moderate to thick data richness and 23 with thin data richness), and moderate concerns about methodological limitation (recruitment, reflexivity, ethics, data collection, and data analysis). |
| 2.5 | **Misconceptions about supplements.** Women, community members, and some health workers and lay health workers had misconceptions about supplements that limited women from taking them to prevent and/or treat anaemia in pregnancy. Common misconceptions included that supplements would increase the size of the fetus and result in women having a difficult childbirth, were only needed to treat women with anaemia, could cause miscarriage, were bad for the fetus or woman’s health, or increased maternal blood and therefore bleeding during birth. These misconceptions were often told to women by family members (e.g., mother-in-law). | [35, 37, 43, 46, 47, 50, 52, 57, 59, 60, 62, 63, 65, 85, 86, 90, 92-94] | Serious concerns: 2 papers with no or very minor concerns, 6 papers with minor concerns (recruitment, reflexivity), 6 papers with moderate concerns (recruitment, reflexivity, data collection, data analysis), and 5 papers with serious concerns (recruitment, reflexivity, ethics, data analysis). | No or very minor concerns | Minor concerns: 17 papers directly and 2 papers indirectly relevant to review aim. Contributing papers represented 4 regions, Africa (Nigeria, Tanzania, Kenya, South Africa, Malawi, Burkina Faso, Ghana), South-East Asia (Bangladesh, Indonesia, India, Nepal), Americas (Bolivia, Guatemala, Honduras), and Eastern Mediterranean (Pakistan), 15 countries where 3 are upper-middle income countries, 9 are lower-middle income countries, and 3 are low-income countries. Perspectives came from pregnant women, non-pregnant women, partner/family members, health workers, lay health workers, traditional birth attendants, and cultural/religious/community leaders. | Moderate concerns: 19 out of 61 papers contributed to the review finding (8 papers with moderate to thick data and 11 papers with thin data). | Moderate confidence | No or very minor concerns about coherence, minor concerns about relevance (4 regions; 3 upper-MICs, 9 lower-MICs, and 3 LICs), moderate concerns about adequacy (19 papers; 8 with moderate to thick data richness and 11 with thin data richness), and serious concerns about methodological limitations (recruitment, reflexivity, ethics, data collection, and data analysis). |
| 2.6 | **Challenges to women remembering to take supplements***.* Many women forgot to take supplements consistently, often due to competing daily activities, such as household chores and work, caring for children, being away from home, or taking other medications. | [36, 38, 39, 41, 43, 47, 53, 54, 57, 59, 62, 63, 68, 72-75, 84, 86, 92, 93] | Moderate concerns: 3 papers with no or very minor concerns, 8 papers with minor concerns (recruitment, reflexivity), 7 papers with moderate concerns (recruitment, reflexivity, data analysis), and 3 papers with serious concerns (recruitment, reflexivity, ethics, data analysis). | No or very minor concerns | No or very minor concerns: 20 papers directly and 1 paper indirectly relevant to review aim. Contributing papers represented 5 regions, Africa (Ethiopia, Tanzania, Zimbabwe, Kenya, South Africa, Zimbabwe, Burkina Faso, Malawi, Nigeria), South-East Asia (India, Thailand, Indonesia), Americas (Bolivia, Guatemala, Honduras), Eastern Mediterranean (Pakistan), and Europe (Ireland), 17 countries where 1 is a high-income country, two are upper-middle income countries, 9 are lower-middle income countries, and 5 are low-income countries. Perspectives came from pregnant women, non-pregnant women, health workers, and lay health workers. | Moderate concerns: 21 out of 61 papers contributed to the review finding (5 papers with moderate data and 16 papers with thin data). | Moderate confidence | No or very minor concerns about coherence and relevance, moderate concerns about adequacy (21 papers; 5 with moderate to thick data richness and 16 with thin data richness), and moderate concerns about methodological limitations (recruitment, reflexivity, ethics, and data analysis). |
| 2.7 | **Inadequate counselling from health workers and lay health workers about supplements.** Women received insufficient and/or inconsistent information on – why they were provided with the supplements, the benefits of taking supplements, when they should start taking supplements, how often they should take supplements, potential side-effects of supplements, and how side-effects were minimised. | [35, 38, 43, 47, 54, 57, 58, 60, 62, 73-75, 86, 89, 90, 93] | Moderate concerns: 2 papers with no or very minor concerns, 6 papers with minor concerns (recruitment, reflexivity), 6 papers with moderate concerns (recruitment, reflexivity, data collection, data analysis), and 2 papers with serious concerns (recruitment, reflexivity, ethics, data analysis). | No or very minor concerns | Minor concerns: 14 papers directly and 2 papers indirectly relevant to review aim. Contributing papers represented 4 regions, Africa (Ethiopia, Burkina Faso, Malawi, Tanzania, Kenya, South Africa, Zimbabwe), South-East Asia (India, Indonesia, Thailand, Cambodia), Americas (Bolivia, Guatemala, Honduras, Argentina), and Eastern Mediterranean (Pakistan), 16 countries where 3 are upper-middle income countries, 7 are lower-middle income countries, and 6 are low-income countries. Perspectives came from pregnant women, non-pregnant women, health workers, lay health workers, traditional birth attendants, and cultural/religious/community leaders. | Minor concerns: 16 out of 61 papers contributed to the review finding (11 papers with moderate to thick data and 5 papers with thin data). | Moderate confidence | No or very minor concerns about coherence, minor concerns about adequacy (16 papers; 11 with moderate to thick data richness and 5 with thin data richness), minor concerns about relevance (4 regions, 3 upper-MICs, 7 lower-MICs, and 6 LICs), and moderate concerns about methodological limitations (recruitment, reflexivity, ethics, data collection, and data analysis). |
| 2.8 | **Insufficient supply of supplements.** Many health workers, lay health workers, and some women described an insufficient supply of supplements, which resulted in health workers and lay health workers prioritising the distribution of supplements to anaemic pregnant women over non-anaemic pregnant women, providing women with an inadequate quantity of supplements, or referring women to other facilities to purchase supplements out-of-pocket (e.g., private drug stores). | [35, 41, 45, 46, 49, 55, 59, 60, 62, 64, 69, 71, 73, 75, 77, 85, 86, 91, 93] | Moderate concerns: 2 papers with no or very minor concerns, 8 papers with minor concerns (recruitment, reflexivity), 7 papers with moderate concerns (recruitment, reflexivity, data collection, data analysis), and 2 papers with serious concerns (recruitment, reflexivity, ethics, data collection, data analysis). | No or very minor concerns | Minor concerns: 16 papers directly and 3 papers indirectly relevant to review aim. Contributing papers represented 4 regions, Africa (Kenya, Ethiopia, Burkina Faso, Malawi, Tanzania, Uganda, Nigeria), South-East Asia (Bangladesh, India, Indonesia), Americas (Bolivia, Guatemala, Honduras), and Eastern Mediterranean (Pakistan), 14 countries where 10 are lower-middle income countries and 4 are low-income countries. Perspectives came from pregnant women, non-pregnant women, partner/family members, health workers, lay health workers, traditional birth attendants, and cultural/religious/community leaders. | Minor concerns: 19 out of 61 papers contributed to the review finding (10 papers with moderate to thick data and 9 papers with thin data). | Moderate confidence | No or very minor concerns about coherence, minor concerns about adequacy (19 papers; 10 with moderate to thick data richness and 9 with thin data richness), minor concerns about relevance (4 regions; 10 lower-MICs and 4 LICs), and moderate concerns about methodological limitations (recruitment, reflexivity, ethics, data collection, and data analysis). |
| 2.9 | **Women's knowledge of supplements and experience of positive benefits.** Adherence to supplements was motivated by women’s knowledge of their health benefits, particularly for the fetus, and for the prevention of illness and excessive blood loss during childbirth. Some women discussed how the relief of anaemia symptoms after taking supplements (e.g., no longer feeling weak or lightheaded) improved adherence. | [36, 38, 41, 47, 52, 53, 60, 62, 68, 73-75, 85, 86, 88, 90-94] | Moderate concerns: 1 paper with no or very minor concerns, 8 papers with minor concerns (recruitment, reflexivity, ethics), 5 papers with moderate concerns (recruitment, reflexivity, data analysis), and 6 papers with serious concerns (recruitment, reflexivity, ethics, data analysis). | No or very minor concerns | No or very minor concerns: 19 papers directly and 1 paper indirectly relevant to review aim. Contributing papers represented 5 regions, Africa (Ethiopia, Nigeria, Burkina Faso, Malawi, Tanzania, Zimbabwe, Kenya, South Africa), South-East Asia (Bangladesh, India, Indonesia, Nepal), Americas (Bolivia, Guatemala, Honduras, Argentina), Eastern Mediterranean (Pakistan), and Europe (Ireland), 18 countries where 1 is a high-income country, 2 are upper-middle income countries, 11 are lower-middle income countries, and 4 are low-income countries. Perspectives came from pregnant women, non-pregnant women, partner/family members, health workers, lay health workers, traditional birth attendants, cultural/religious/community leaders, and non-governmental organisation staff. | Minor concerns: 20 out of 61 papers contributed to the review finding (11 papers with moderate to thick data and 9 papers with thin data). | Moderate confidence | No or very minor concerns about coherence and relevance, minor concerns about adequacy (20 papers; 11 with moderate to thick data richness and 9 with thin data richness), and moderate concerns about methodological limitations (recruitment, reflexivity, ethics, and data analysis). |
| 2.10 | **Influence of family support on women taking supplements.** Many women, health workers, lay health workers, and community members highlighted how women's adherence to supplements was influenced by whether they had received encouragement and/or reminders to take supplements from family members (most commonly their husband, mother-in-law, or parents). | [38, 39, 43, 47, 50-52, 57, 63, 66, 68, 73, 74, 76, 82, 86, 92, 94] | Moderate concerns: 1 paper with no or very minor concerns, 8 papers with minor concerns (recruitment, reflexivity), 5 papers with moderate concerns (recruitment, reflexivity, data analysis), and 4 papers with serious concerns (recruitment, reflexivity, ethics, data analysis). | No or very minor concerns | Minor concerns: 17 papers directly and 1 paper indirectly relevant to review aim. Contributing papers represented 3 regions, Africa (Ethiopia, Nigeria, Kenya, South Africa, Zimbabwe), South-East Asia (Indonesia, India, Nepal), and Eastern Mediterranean (Pakistan), 9 countries where 1 is an upper-middle income country, 5 are lower-middle income countries, and 3 are low-income countries. Perspectives came from pregnant women, non-pregnant women, partner/family members, health workers, lay health workers, traditional birth attendants, cultural/religious/community leaders, and non-governmental organisation staff. | Minor concerns: 18 out of 61 papers contributed to the review finding (11 papers with moderate to thick data and 7 papers with thin data). | Moderate confidence | No or very minor concerns about coherence, minor concerns about adequacy (18 papers; 11 with moderate to thick data richness and 7 with thin data richness), minor concerns about relevance (3 regions; 1 upper-MIC, 5 lower-MICs, and 3 LICs), and moderate concerns about methodological limitations (recruitment, reflexivity, ethics, and data analysis). |
| 2.11 | **Reminder strategies to take supplements.** Some women and health workers perceived reminder strategies as facilitating women’s adherence to supplements. Reminder strategies included linking taking supplements with other daily behaviour (e.g., with mealtime) and storing supplements in a visible location. | [36, 57, 73, 75] | Minor concerns: 3 papers with minor concerns (recruitment, reflexivity), 1 paper with moderate concerns (reflexivity, data analysis). | No or very minor concerns | Serious concerns: All 4 papers directly relevant to review aim. Contributing papers represented 3 regions, Africa (Ethiopia, Kenya), South-East Asia (India), and Europe (Ireland), 4 countries where 1 is a high-income country, 2 are lower-middle income countries, and 1 is a low-income country. Perspectives came from pregnant women, non-pregnant women, health workers, lay health workers, and traditional birth attendants. | Serious concerns: 4 out of 61 papers contributed to the review finding (1 paper with moderate data and 3 papers with thin data). | Very low confidence | No or very minor concerns about coherence, minor concerns about methodological limitations (recruitment, reflexivity, and data analysis), serious concerns about adequacy (4 papers; 1 with moderate data richness and 3 with thin data richness), and serious concerns about relevance (3 regions; 1 HIC, 2 lower-MICs and 1 LIC). |
| 2.12 | **Recommendation from health workers to take supplements.** Many women reported taking supplements because they trusted and accepted the advice of health workers. | [38, 41, 52, 57, 59, 60, 62, 63, 68, 73, 74, 85-88, 94] | Moderate concerns: 1 paper with no or very minor concerns, 8 papers with minor concerns (recruitment, reflexivity, ethics), 5 papers with moderate concerns (recruitment, reflexivity, data analysis), and 2 papers with serious concerns (recruitment, reflexivity, ethics, data analysis). | No or very minor concerns | Minor concerns: All 16 papers directly relevant to review aim. Contributing papers represented 3 regions, Africa (Ethiopia, Burkina Faso, Nigeria, Tanzania, Kenya, Zimbabwe), South-East Asia (Bangladesh, India, Nepal, Indonesia), and Eastern Mediterranean (Pakistan), 11 countries where 1 is an upper-middle income country, 6 are lower-middle income countries and 4 are low-income countries. Perspectives came from pregnant women and non-pregnant women. | Minor concerns: 16 out of 61 papers contributed to the review finding (6 papers with moderate to thick data and 10 papers with thin data). | Moderate confidence | No or very minor concerns about coherence, minor concerns about adequacy (16 papers; 6 with moderate to thick data richness and 10 with thin data richness), minor concerns about relevance (3 regions; 1 upper-MIC, 6 lower-MICs and 4 LICs), and moderate concerns about methodological limitations (recruitment, reflexivity, ethics, and data analysis). |
| 2.13 | **Alternative distribution methods for supplements.** Most women, health workers, and lay health workers supported distributing supplements to women during home visits. Lay health workers discussed how women's adherence to supplements improved with home visits, as they also allowed for health education and counselling. Some women discussed how community-based distribution mitigated barriers to receiving supplements, such as distance to health facilities and transportation costs. | [47, 64, 73, 89] | Minor concerns: 2 papers with no or very minor concerns, 1 paper with minor concerns (reflexivity), and 1 paper with moderate concerns (reflexivity, data analysis). | No or very minor concerns | Serious concerns: 3 papers directly and 1 paper indirectly relevant to review aim. Contributing papers represented 2 regions, Africa (Ethiopia, Kenya, South Africa) and South-East Asia (Cambodia), 4 countries where 1 is an upper-middle income country, 1 is a lower-middle income country, and 2 are low-income countries. Perspectives came from pregnant women, non-pregnant women, health workers, and lay health workers. | Serious concerns: 4 out of 61 papers contributed to the review finding (3 papers with moderate to thick data and 1 paper with thin data). | Very low confidence | No or very minor concerns about coherence, minor concerns about methodological limitations (reflexivity and data analysis), serious concerns about adequacy (4 papers; 3 with moderate to thick data richness and 1 with thin data richness), and serious concerns about relevance (2 regions; 1 upper-MIC, 1 lower-MIC, and 2 LICs). |
| 2.14 | **Blood transfusion as treatment for severe anaemia in pregnant women.** Women and health workers perceived blood transfusion as treatment for women with severe anaemia; however, cost, religion, blood and donor availability, fear of contracting diseases, and a woman’s social position limited access. | [34, 39, 45, 52, 54, 57, 80, 87, 93] | Moderate concerns: 2 papers with no or very minor concerns, 6 papers with minor concerns (reflexivity, data analysis), and 1 paper with serious concerns (reflexivity, ethics, data analysis). | No or very minor concerns | Moderate concerns: All 9 papers directly relevant to review aim. Contributing papers represented 3 regions, South-East Asia (India, Thailand, Nepal), Africa (Burkina Faso, Malawi, Nigeria, Ghana, Uganda), and Western Pacific (Australia), 9 countries where 1 is a high-income country, 1 is an upper-middle income country, 4 are lower-middle income countries, and 3 are low-income countries. Perspectives came from pregnant women, non-pregnant women, partners/family members, and health workers. | Serious concerns: 9 out of 61 papers contributed to the review finding (2 papers with moderate to thick data and 7 papers with thin data). | Low confidence | No or very minor concerns about coherence, moderate concerns about methodological limitations (reflexivity, ethics, and data analysis), moderate concerns about relevance (3 regions; 1 HIC, 1 upper-MIC, 4 lower-MICs, and 3 LICs), and serious concerns about adequacy (9 papers; 2 with moderate to thick data richness and 7 with thin data richness). |
| 2.15 | **Intravenous iron as an alternative treatment option for iron-deficiency anaemia in pregnant women.** Health workers, women, and community members described intravenous iron as a treatment option for women who had iron-deficiency anaemia and were unable to take supplements (e.g., due to side-effects), who attended antenatal care late, or who were unable to have a blood transfusion because of religious beliefs. | [39, 40, 48, 56, 57, 80, 90] | Moderate concerns: 5 papers with minor concerns (recruitment, reflexivity, data collection, data analysis), 1 paper with moderate concerns (reflexivity, data analysis), and 1 paper with serious concerns (recruitment, reflexivity, ethics, data analysis). | No or very minor concerns | Moderate concerns: All 7 papers directly relevant to review aim. Contributing papers represented 5 regions, Africa (Nigeria, Malawi), South-East Asia (India), Americas (Argentina), Western Pacific (Australia), and Eastern Mediterranean (Pakistan), 6 countries where one is a high-income country, 1 is an upper-middle income country, 3 are lower-middle income countries, and 1 is a low-income country. Perspectives came from pregnant women, non-pregnant women, partners/family members, and health workers. | Serious concerns: 7 out of 61 papers contributed to the review finding (4 papers with thick data and 3 papers with thin data). | Low confidence | No or very minor concerns about coherence, moderate concerns about methodological limitations (recruitment, reflexivity, ethics, data collection, and data analysis), moderate concerns about relevance (5 regions; 1 HIC, 1 upper-MIC, 3 lower-MICs, and 1 LIC), and serious concerns about adequacy (7 papers; 4 with thick data richness and 3 with thin data richness). |
|  | **3.0 Testing pregnant women for anaemia** | | | | | | | |
| 3.1 | **Benefits of testing pregnant women for anaemia with point-of-care devices using capillary blood.** Health workers provided with anaemia point-of-care devices (portable haemoglobinometers or a colour scale) felt they were easy to use with capillary blood and were eager to meet pregnant women's antenatal care needs. Health workers described how more women were diagnosed with anaemia as they were no longer lost to follow-up during the referral process to laboratories for a full blood count using venous blood. Women described how haemoglobin results from a point-of-care test improved their trust in the anaemia diagnosis and increased their involvement in the treatment decision-making process. | [61, 69] | Minor concerns: 2 papers with minor concerns (reflexivity). | No or very minor concerns | Serious concerns: 1 paper directly and 1 paper indirectly relevant to review aim. Contributing papers represented 1 region, Africa (Ghana, Kenya), 2 countries where 2 are lower-middle income countries. Perspectives came from pregnant women, health workers, and laboratory staff. | Serious concerns: 2 out of 61 papers contributed to the review finding (2 papers with thick data). | Very low confidence | No or very minor concerns about coherence, minor concerns about methodological limitations (reflexivity), serious concerns about adequacy (2 papers; 2 with thick data richness), and serious concerns about relevance (1 region; 2 lower-MICs). |
| 3.2 | **Challenges to testing pregnant women for anaemia with point-of-care devices using capillary blood and full blood count using venous blood.** Challenges to health workers using anaemia point-of-care devices with capillary blood included a shortage of tests, staff, and equipment (e.g., cuvettes required for most haemoglobinometers), inadequate training, and heavy workloads. For full blood count using venous blood, challenges described by health workers included a lack of laboratory facilities and women not attending facilities when referred. Health workers expressed reservations about the accuracy of results for both types of anaemia tests. | [34, 45, 46, 61, 65, 67, 69, 75, 77, 82, 83] | Minor concerns: 1 paper with no or very minor concerns, 8 papers with minor concerns (recruitment, reflexivity) and 2 papers with moderate concerns (recruitment, reflexivity). | No or very minor concerns | Minor concerns: 9 papers directly and 2 papers indirectly relevant to review aim. Contributing papers represented 2 regions, South-East Asia (Indonesia, India) and Africa (Tanzania, Uganda, Ghana, Kenya, Nigeria), 7 countries where 1 is an upper-middle income country, 5 are lower-middle income countries, and 1 is a low-income country. Perspectives came from health workers, lay health workers, laboratory staff, and non-governmental organisation staff. | Moderate concerns: 11 out of 61 papers contributed to the review finding (7 papers with thick data and 4 papers with thin data). | Low confidence | No or very minor concerns about coherence, minor concerns about methodological limitations (recruitment and reflexivity), minor concerns about relevance (2 regions; 1 upper-MIC, 5 lower-MICs, and 1 LIC), and moderate concerns about adequacy (11 papers; 7 with thick data richness and 4 with thin data richness). |
|  | **4.0 Factors affecting health workers’ engagement in the management of anaemia in pregnant women** | | | | | | | |
| 4.1 | **Training and guidance on how to manage anaemia in pregnant women among health workers and lay health workers.** Some health workers and lay health workers described receiving inadequate training on how to manage anaemia in pregnant women (e.g., on counselling, supplements, and/or testing). They also described a lack of specific or standardised guidelines on how to manage anaemia in pregnant women. | [34, 45, 49, 61, 62, 64, 65, 67, 69, 71, 73, 75, 77, 80, 82, 85] | Minor concerns: 2 papers with no or very minor concerns, 8 papers with minor concerns (reflexivity), and 6 papers with moderate concerns (recruitment, reflexivity, data analysis). | No or very minor concerns | Minor concerns: 13 papers directly and 3 papers indirectly relevant to review aim. Contributing papers represented 3 regions, Africa (Ethiopia, Kenya, Uganda, Tanzania, Ghana, Nigeria), South-East Asia (Bangladesh, Indonesia, India), and Western Pacific (Australia), 10 countries where 1 is a high-income country, 1 is an upper-middle income country, 6 are lower-middle income countries, and 2 are low-income countries. Perspectives came from health workers, lay health workers, traditional birth attendants, and laboratory staff. | Minor concerns: 16 out of 61 papers contributed to the review finding (8 papers with moderate to thick data and 8 papers with thin data). | Moderate confidence | No or very minor concerns about coherence, minor concerns about methodological limitations (recruitment, reflexivity, and data analysis), minor concerns about adequacy (16 papers; 8 with moderate to thick data richness and 8 with thin data richness), and minor concerns about relevance (3 regions; 1 HIC, 1 upper-MIC, 6 lower-MICs, and 2 LICs). |
| 4.2 | **Health worker and lay health worker challenges to managing anaemia in pregnant women.** Many health workers and lay health workers described how staffing shortages, heavy workloads, insufficient facilities and space, and poor work conditions affected their ability to manage anaemia in pregnant women. | [34, 45, 55, 57, 60, 62, 65, 67, 69, 71, 73, 77, 82, 83] | Minor concerns: 1 paper with no or very minor concerns, 9 papers with minor concerns (recruitment, reflexivity), and 4 papers with moderate concerns (recruitment, reflexivity, data analysis). | No or very minor concerns | Minor concerns: 13 papers directly and 1 paper indirectly relevant to review aim. Contributing papers represented 2 regions, Africa (Kenya, Tanzania, Uganda, Ethiopia, Nigeria, Ghana) and South-East Asia (Indonesia, India), 8 countries where 1 is an upper-middle income country, 5 are lower-middle income countries, and 2 are low-income countries. Perspectives came from health workers, lay health workers, and laboratory staff. | Minor concerns: 14 out of 61 papers contributed to the review finding (7 papers with moderate to thick data and 7 papers with thin data). | Moderate confidence | No or very minor concerns about coherence, minor concerns about methodological limitations (recruitment, reflexivity, and data analysis), minor concerns about adequacy (14 papers; 7 with moderate to thick data richness and 7 with thin data richness), and minor concerns about relevance (2 regions; 1 upper-MIC, 5 lower-MICs, and 2 LICs). |
