# Supplementary material for: Perceptions and experiences of the prevention, testing, and treatment of anaemia in pregnant women: A qualitative evidence synthesis
Source: PLOS Glob Public Health. 2025 Oct 1;5(10):e0005158. doi: 10.1371/journal.pgph.0005158 (PMC12488017; doi:10.1371/journal.pgph.0005158)
Supplement: S4 Appendix — (DOCX) [file pgph.0005158.s004.docx]

**S4 Appendix: Characteristics of included papers**

|  | | | | | | **Topic** | | | |
| --- | --- | --- | --- | --- | --- | --- | --- | --- | --- |
| **Author** | **Title** | **Country** | **Data collection method** | **Participants** | **Sample size** | **Diet** | **Supplementation** (iron, iron and folic acid, and/or multiple micronutrient) | **Clinical intervention** (blood transfusion and/or  intravenous iron) | **Testing**  (point-of-care devices using capillary blood and/or full blood count using venous blood) |
| Abebaw 2020 | Adherence to iron and folic acid supplementation and associated factors among antenatal care attendants in Northwest Ethiopia | Ethiopia | Interviews | Pregnant women  Health workers | Pregnant women: 6  Health workers: 2 |  |  |  |  |
| Akinajo 2024 | Implementation fidelity of intravenous ferric carboxymaltose administration for iron-deficiency anaemia in pregnancy: a mixed-methods study nested in a clinical trial in Nigeria | Nigeria | Interviews | Health workers | 14 |  |  |  |  |
| Akinajo 2024 | Acceptability of IV iron treatment for iron deficiency anaemia in pregnancy in Nigeria: a qualitative study with pregnant women, domestic decision-makers, and health care providers | Nigeria | Interviews  Focus group discussions | Pregnant women  Partner/family members  Health workers  Laboratory staff | Interviews: 29  Focus group discussions: 140 |  |  |  |  |
| Alam 2015 | How can formative research inform the design of an iron-folic acid supplementation intervention starting in first trimester of pregnancy in Bangladesh? | Bangladesh | Interviews | Pregnant women  Non-pregnant women  Partner/family members  Lay health workers  Traditional birth attendants  Health workers | 86 |  |  |  |  |
| Aziz Ali 2021 | Perceptions of women, their husbands and healthcare providers about anemia in rural Pakistan: Findings from a qualitative exploratory study | Pakistan | Interviews  Focus group discussions | Non-pregnant women  Partner/family members  Health workers  Traditional birth attendants | 113 |  |  |  |  |
| AregaSadore 2015 | Compliance with iron-folate supplement and associated factors among antenatal care attendant mothers in Misha District, South Ethiopia: Community Based Cross-Sectional Study | Ethiopia | Interviews | Pregnant women  Lay health workers | Pregnant women: 8  Lay health workers: 5 |  |  |  |  |
| Atmadani 2024 | Supports and barriers regarding the iron-folic acid supplementation adherence level in anemic pregnant women: Indonesian’s perspective | Indonesia | Interviews | Pregnant women | 25 |  |  |  |  |
| Bahati 2021 | Adherence to iron and folic acid supplementation during pregnancy among postnatal mothers seeking maternal and child healthcare at Kakamega level 5 hospital in Kenya: a cross-sectional study | Kenya | Interviews | Health workers  Lay health workers | Health workers: 5  Lay health workers: 11 |  |  |  |  |
| Baker 2015 | Bottlenecks in the implementation of essential screening tests in antenatal care: Syphilis, HIV, and anemia testing in rural Tanzania and Uganda | Tanzania  Uganda | Interviews | Health workers | 15 |  |  |  |  |
| Baker 2020 | Antenatal testing for anaemia, HIV and syphilis in Indonesia - a health systems analysis of low coverage | Indonesia | Interviews  Focus group discussions | Lay health workers  Health workers  Laboratory staff  Non-governmental organisational staff | 40 |  |  |  |  |
| Birhanu 2016 | Ethiopian women's perspectives on antenatal care and iron-folic acid supplementation: Insights for translating global antenatal calcium guidelines into practice | Ethiopia | Interviews | Health workers (nurses)  Lay health workers  Traditional birth attendants  Pregnant women | Health workers, Lay health workers, and Traditional birth attendants: 22  Pregnant women: 20 |  |  |  |  |
| Chatterjee 2014 | 'This is normal during pregnancy': A qualitative study of anaemia-related perceptions and practices among pregnant women in Mumbai, India | India | Interviews  Focus group discussions | Pregnant women | 31 |  |  |  |  |
| Compaore 2014 | "There is iron and iron..." Burkinabe women's perceptions of iron supplementation: A qualitative study | Burkina Faso | Focus group discussions | Non-pregnant women  Lay health worker | 100 to 120 |  |  |  |  |
| Darmawati 2020 | Acehnese Cultural Leaders' perspective on anemia in pregnant women: A qualitative study | Indonesia | Interviews  Focus group discussions | Cultural/religious/community leaders | 12 |  |  |  |  |
| Darmawati 2020 | Barriers to Health Workers in Iron Deficiency Anemia Prevention among Indonesia Pregnant Women | Indonesia | Interviews | Health workers | 18 |  |  |  |  |
| Darmawati 2022 | Husband's Perception on Anemia among Pregnant Women based on Cultural Perspective: A Qualitative Study | Indonesia | Interviews | Partner/family members | 12 |  |  |  |  |
| Darmawati 2022 | Exploring Indonesian mothers' perspective on anemia during pregnancy: A qualitative approach | Indonesia | Focus group discussions | Pregnant women | 24 |  |  |  |  |
| Diamond-Smith 2016 | Determinants of persistent anemia in poor, urban pregnant women of Chandigarh city, North India: A mixed method approach | India | Focus group discussions | Pregnant women  Health workers  Lay health workers | Pregnant women: 11  Health workers and lay health workers: 6 |  |  |  |  |
| Ejidokun 2000 | Community attitudes to pregnancy, anaemia, iron and folate supplementation in urban and rural Lagos, south-western Nigeria | Nigeria | Interviews  Focus group discussions | Pregnant women  Health workers | Pregnant women: 23  Health workers: 2 |  |  |  |  |
| Ferka 2024 | Pregnant women’s perceptions of daily iron supplementation in rural Ghana | Ghana | Interviews | Pregnant women | 20 |  |  |  |  |
| Galloway 2002 | Women's perceptions of iron deficiency and anaemia prevention and control in eight developing countries | Bolivia  Burkina Faso  Guatemala  Honduras  India  Indonesia  Malawi  Pakistan | Interviews  Focus group discussions  Observations  Social mapping  Case studies | Pregnant women  Non-pregnant women  Health workers  Partner/family members  Lay health workers  Cultural/religious/community leaders | See table 1 in paper |  |  |  |  |
| Getachew 2018 | Magnitude and factors associated with adherence to iron-folic acid supplementation among pregnant women in Eritrean refugee camps, northern Ethiopia | Ethiopia | Interviews  Focus group discussions | Pregnant women  Health workers | Pregnant women: 56  Health workers: 3 |  |  |  |  |
| Ghanekar 2002 | Toward better compliance with iron-folic acid supplements: Understanding the behaviour of poor urban pregnant women through ethnographic decision models in Vadodara, India | India | Interviews | Pregnant women  Partner/family members | Pregnant women: 36  Partner/family members: 20 |  |  |  |  |
| Gillespie 2023 | Attention for and awareness of anemia in adolescents in Karnataka, India: A qualitative study | India | Interviews  Focus group discussions | Non-pregnant women  Health workers  Partner/family members  Cultural/religious/community leaders | 64 |  |  |  |  |
| Kamau 2020 | Time for change is now: Experiences of participants in a community-based approach for iron and folic acid supplementation in a rural country in Kenya, a qualitative study | Kenya | Interviews | Health workers  Lay health workers  Pregnant women | Health workers: 2  Lay health workers: 9  Pregnant women: 8 |  |  |  |  |
| Klankhajhon 2021 | Perspectives of pregnant women regarding iron deficiency anaemia | Thailand | Interviews | Pregnant women | 18 |  |  |  |  |
| Kuliya-Gwarzo 2023 | Maternal anaemia care in Kano state, Nigeria: an exploratory qualitative study of experiences of uptake and provision | Nigeria | Interviews  Focus group discussions | Health workers  Lay health workers  Pregnant women  Non-pregnant women  Laboratory staff | 56 |  |  |  |  |
| Lacerte 2011 | Determinants of adherence to iron/folate supplementation during pregnancy in two provinces in Cambodia | Cambodia | Interviews | Non-pregnant women  Pregnant women | Non-pregnant women: 10  Pregnant women: 10 |  |  |  |  |
| Lavanya 2020 | Adherence to iron and folic acid supplementation among antenatal mothers attending a tertiary care center Puducherry: A mixed-methods study | India | Interviews | Pregnant women  Health workers | Pregnant women: 8  Health workers: 4 |  |  |  |  |
| Luwangula 2022 | Improving iron and folic acid supplementation among pregnant women: An implementation science approach in east-central Uganda | Uganda | Interviews | Health workers | 26 |  |  |  |  |
| Lyoba 2020 | Adherence to Iron-Folic Acid Supplementation and Associated Factors among Pregnant Women in Kasulu Communities in North-Western Tanzania | Tanzania | Interviews  Focus group discussions | Health workers  Pregnant women  Non-pregnant women | Health workers: 6  Pregnant women: 19  Non-pregnant women: 15 |  |  |  |  |
| Mahundi 2021 | Barriers to optimal iron supplementation by pregnant women attending the Mutare City Clinic, Manicaland, Zimbabwe | Zimbabwe | Focus group discussions | Pregnant women | 64 |  |  |  |  |
| Manda-Taylor 2022 | Perceptions and experiences of intravenous iron treatment for anaemia in pregnancy in Malawi: a formative qualitative study | Malawi | Interviews  Focus group discussions | Pregnant women  Health workers | Interviews: 15  Focus group discussions: 14 |  |  |  |  |
| Martin 2017 | Adherence partners are an acceptable behaviour change strategy to support calcium and iron-folic acid supplementation among pregnant women in Ethiopia and Kenya | Ethiopia and Kenya | Interviews | Pregnant women | 35 |  |  |  |  |
| Martin 2017 | Translating formative research findings into a behaviour change strategy to promote antenatal calcium and iron and folic acid supplementation in western Kenya | Kenya | Interviews | Pregnant women  Non-pregnant women  Health workers  Lay health workers  Traditional birth attendants | Pregnant women: 13  Non-pregnant women: 9  Health workers: 8  Lay health workers: 7  Traditional birth attendants: 5 |  |  |  |  |
| Mayson 2016 | Intravenous iron: barriers and facilitators to its use at nine maternity hospitals in New South Wales, Australia | Australia | Interviews | Health workers | 61 |  |  |  |  |
| Morrison 2021 | Addressing anaemia in pregnancy in rural plains Nepal: A qualitative, formative study | Nepal | Interviews  Focus group discussions | Pregnant women  Partner/family members  Health workers  Lay health workers  Cultural/religious/community leaders  Non-governmental organisational staff | Pregnant women: 16  Partner/family members: 39  Health workers: 4 |  |  |  |  |
| Morrison 2023 | Assessing food-based strategies to address anaemia in pregnancy in rural plains Nepal: a mixed methods study | Nepal | Interviews  Focus group discussions | Non-pregnant women  Partner/family members  Health workers  Cultural/religious/community leaders  Lay health workers  Non-governmental organisational staff | 63 |  |  |  |  |
| Muthuraj 2023 | Sociocultural and drug-related factors associated with adherence to iron-folic acid supplementation  among pregnant women – a mixed-methods study | India | Focus group discussions | Pregnant women  Partner/family members  Health workers | 60 to 80 |  |  |  |  |
| Nahrisah 2019 | Micronutrient intake and perceived barriers among anaemic pregnant women in Aceh, Indonesia | Indonesia | Focus group discussions | Pregnant women | 7 |  |  |  |  |
| Nisar 2014 | Perceptions of antenatal iron-folic acid supplements in urban and rural Pakistan: a qualitative study | Pakistan | Interviews  Focus group discussions | Non-pregnant women  Pregnant women  Lay health workers  Health workers | Non-pregnant women: 73  Pregnant women: 10  Lay health workers: 6  Health workers: 4 |  |  |  |  |
| Onyeneho 2016 | Anaemia is typical of pregnancies: capturing community perception and management of anaemia in pregnancy in Anambra State, Nigeria | Nigeria | Interviews  Focus group discussions | Non-pregnant women  Partner/family members  Health workers | Interviews: 20  Focus group discussions: 151 |  |  |  |  |
| Onyeneho 2016 | Factors associated with compliance to recommended micronutrients uptake for prevention of anaemia during pregnancy in urban, peri-urban, and rural communities in Southeast Nigeria | Nigeria | Interviews  Focus group discussions | Partner/family members  Health workers | Interviews: 6  Focus group discussions: 96 to 144 |  |  |  |  |
| O’Toole 2024 | The experience of anaemia and ingesting oral iron supplementation in pregnancy: a qualitative study | Ireland | Interviews | Pregnant women  Non-pregnant women | 14 |  |  |  |  |
| Palmer 2020 | Improving the effectiveness of point of care tests for malaria and anaemia: a qualitative study across three Ghanaian antenatal clinics | Ghana | Interviews  Focus group discussions | Health workers  Laboratory staff  Pregnant women | Health workers and laboratory staff: 11  Pregnant women: 40 |  |  |  |  |
| Pasaribu 2024 | Anaemia in pregnancy: study phenomenology | Indonesia | Interviews  Focus group discussions | Pregnant women  Partner/family members  Health workers  Cultural/religious/community leaders | Interviews: 20  Focus group discussions: 12 |  |  |  |  |
| Riang’a 2020 | Implementation fidelity of nutritional counselling, iron and folic acid supplementation guidelines and associated challenges in rural Uasin Gishu Country Kenya | Kenya | Interviews  Questionnaire | Pregnant women  Health workers | Pregnant women: 188  Health workers: 6 |  |  |  |  |
| Sammartino 2010 ^a^ | Cultural representations about anaemia and supplement with iron | Argentina | Interviews  Observations | Health workers  Non-pregnant women  Pregnant women | Health workers: 49  Non-pregnant women and pregnant women: 30 |  |  |  |  |
| Saraswathy 2023 | Predictors of maternal and fetal outcome in severely anemic pregnant mothers: a sequential mixed methods study | India | Interviews | Pregnant women | 15 |  |  |  |  |
| Sedlander 2020 | Moving beyond individual barriers and identifying multi-level strategies to reduce anemia in Odisha India | India | Interviews  Focus group discussions  Observations of health facilities | Non-pregnant women  Partner/family members  Health workers  Lay health workers | 149  Observations of health facilities: 17 |  |  |  |  |
| Silubonde 2022 | Barriers and facilitators of micronutrient supplementation among non-pregnant women of reproductive age in Johannesburg, South Africa | South Africa | Interviews  Focus group discussions | Lay health workers  Non-pregnant women | 58 |  |  |  |  |
| Tancred 2024 | Prevention and management of anaemic in pregnancy: Community perceptions and facility readiness in Ghana and Uganda | Ghana  Uganda | Interviews  Focus group discussions | Pregnant women  Non-pregnant women  Partner/family members  Cultural/religious/community leaders  Health workers  Laboratory staff | Ghana: 84  Uganda: 63 |  |  |  |  |
| Tefera 2023 | Adherence to iron and folate supplementation and associated factors among women attending antenatal care in public health facilities at COVID-19 pandemic in Ethiopia | Ethiopia | Interviews | Pregnant women  Health workers | Pregnant women: 4  Health workers: 8 |  |  |  |  |
| Tinago 2017 | Individual and structural environmental influences on utilisation of iron and folic acid supplementation among pregnant women in Harare, Zimbabwe | Zimbabwe | Interviews | Pregnant women  Health workers | Pregnant women: 24  Health workers: 14 |  |  |  |  |
| Wana 2020 | Predictors of prenatal iron folic acid supplement utilisation in Wolaita, South Ethiopia: A community based cross-sectional study (quantitative and qualitative approach) | Ethiopia | Interviews  Focus group discussions | Pregnant women  Health workers  Lay health workers | Interviews: 8  Focus group discussions: n/a |  |  |  |  |
| Wendt 2018 | Identifying bottlenecks in the iron and folic acid supply chain in Bihar, India: a mixed-methods study | India | Interviews | Health workers  Lay health workers | 59 |  |  |  |  |
| Widyawati 2015 | A qualitative study on barriers in the prevention of anaemia during pregnancy in public health centres: perceptions of Indonesian nurse-midwives | Indonesia | Interviews | Health workers | 23 |  |  |  |  |
| Widyawati 2016 | Perceived barriers and facilitators of a new model in managing pregnant women with iron deficiency anemia: A qualitative study | Indonesia | Focus group discussions | Health workers | 24 |  |  |  |  |
| Williams 2020 | Strategies to address anaemia among pregnant and lactating women in India: A formative research study | India | Interviews  Focus group discussions | Pregnant women  Non-pregnant women  Health workers | 79 |  |  |  |  |
| Young 2005 | Linking traditional treatments of maternal anaemia to iron supplement use: an ethnographic case study from Pemba Island, Zanzibar | Tanzania | Informal conversations  Interviews  Focus group discussions  Observations | Non-pregnant women  Health workers  Lay health workers  Traditional birth attendants | Non-pregnant women: 25  Health workers, Lay health workers, and Traditional Birth Attendants: 27 |  |  |  |  |
| Young 2019 | Integrated point-of-care testing (POCT) for HIV, syphilis, malaria and anaemia at antenatal facilities in western Kenya: a qualitative study exploring end-users’ perspectives of appropriateness, acceptability and feasibility | Kenya | Interviews  Focus group discussions | Health workers  Pregnant women | Health workers: 18  Pregnant women: 118 |  |  |  |  |

^a^: a study published in Spanish
