# Supplementary material for: Perceptions and experiences of the prevention, testing, and treatment of anaemia in pregnant women: A qualitative evidence synthesis
Source: PLOS Glob Public Health. 2025 Oct 1;5(10):e0005158. doi: 10.1371/journal.pgph.0005158 (PMC12488017; doi:10.1371/journal.pgph.0005158)
Supplement: S5 Appendix — (DOCX) [file pgph.0005158.s005.docx]

**S5 Appendix: Methodological limitations of included studies**

| **Author** | **Research aims** | **Qualitative methodology** | **Research design** | **Recruitment** | **Data collection** | **Reflexivity** | **Ethics** | **Data analysis** | **Support for findings** | **Overall CASP rating** |
| --- | --- | --- | --- | --- | --- | --- | --- | --- | --- | --- |
| **Author** | **Was there a statement of the aims of the research?** | **Given the aim, was a qualitative methodology appropriate?** | **Was the research design appropriate to address the aim?** | **Was the recruitment strategy appropriate to the aims of the research?** | **Was the data collected in a way that addressed the research issue?** | **Was the relationship between the researcher and participants adequately considered?** | **Have ethical issues been taken into consideration?** | **Was the data analysis sufficiently rigorous?** | **Were the findings supported by the evidence?** | **Overall assessment of methodological limitations** |
| Abebaw  2020 | Yes | Yes | Yes | No | Yes | No | Yes | Partial | Yes | Serious concerns |
| Akinajo  2024 ^a^ | Yes | Yes | Yes | Yes | Partial | No | Yes | Yes | Yes | Minor concerns |
| Akinajo  2024 ^b^ | Yes | Yes | Yes | Yes | Yes | No | Yes | Yes | Yes | Minor concerns |
| Alam  2015 | Yes | Yes | Yes | Yes | Yes | Partial | Yes | Partial | Yes | Moderate concerns |
| Aziz Ali  2021 | Yes | Yes | Yes | Yes | Yes | Yes | Yes | Partial | Yes | Minor concerns |
| AregaSadore 2015 | Yes | Yes | Yes | Yes | Yes | No | Yes | Partial | Yes | Moderate concerns |
| Atmadani  2024 | Yes | Yes | Yes | Partial | Yes | No | Yes | Partial | Yes | Moderate concerns |
| Bahati  2021 | Yes | Yes | Yes | Yes | Yes | No | Yes | Partial | Yes | Moderate concerns |
| Baker  2015 | Yes | Yes | Yes | Yes | Yes | No | Yes | Yes | Yes | Minor concerns |
| Baker  2020 | Yes | Yes | Yes | Yes | Yes | No | Yes | Yes | Yes | Minor concerns |
| Birhanu  2016 | Yes | Yes | Yes | Yes | Yes | No | Yes | Yes | Yes | Minor concerns |
| Chatterjee  2014 | Yes | Yes | Yes | Yes | Yes | Partial | Partial | Yes | Yes | Minor concerns |
| Compaore  2014 | Yes | Yes | Yes | Partial | Yes | No | Yes | Yes | Yes | Minor concerns |
| Darmawati ^c^ 2020 | Yes | Yes | Yes | No | Yes | No | Yes | Yes | Yes | Moderate concerns |
| Darmawati ^d^ 2020 | Yes | Yes | Yes | Yes | Yes | No | Yes | Yes | Yes | Minor concerns |
| Darmawati ^e^ 2022 | Yes | Yes | Yes | Yes | Yes | No | No | Yes | Yes | Serious concerns |
| Darmawati ^f^ 2022 | Yes | Yes | Yes | Yes | Yes | No | Yes | Yes | Yes | Minor concerns |
| Diamond-Smith  2016 | Yes | Yes | Yes | Partial | Yes | No | Yes | Partial | Yes | Moderate concerns |
| Ejidokun  2000 | Yes | Yes | Yes | Partial | Yes | No | No | Partial | Yes | Serious concerns |
| Ferka  2024 | Yes | Yes | Yes | Yes | Partial | No | Yes | Partial | Yes | Moderate concerns |
| Galloway  2002 | Yes | Yes | Yes | Yes | Yes | No | No | Partial | Yes | Serious concerns |
| Getachew  2018 | Yes | Yes | Yes | Yes | Yes | No | Yes | Yes | Yes | Minor concerns |
| Ghanekar  2002 | Yes | Yes | Yes | Partial | Yes | No | No | Partial | Yes | Serious concerns |
| Gillespie  2023 | Yes | Yes | Yes | Partial | Yes | Partial | Yes | Yes | Yes | Minor concerns |
| Kamau  2020 | Yes | Yes | Yes | Partial | Yes | Yes | Yes | Yes | Yes | No or very minor concerns |
| Klankhajhon 2021 | Yes | Yes | Yes | Yes | Yes | Yes | Yes | Yes | Yes | No or very minor concerns |
| Kuliya-Gwarzo 2023 | Yes | Yes | Yes | Yes | Yes | No | Yes | Yes | Yes | Minor concerns |
| Lacerte  2011 | Yes | Yes | Yes | Yes | Yes | No | Yes | Partial | Yes | Moderate concerns |
| Lavanya  2020 | Yes | Yes | Yes | Partial | Yes | No | Yes | Partial | Yes | Moderate concerns |
| Luwangula  2022 | Yes | Yes | Yes | Partial | Yes | No | Yes | Partial | Yes | Moderate concerns |
| Lyoba  2020 | Yes | Yes | Yes | Yes | Yes | No | Yes | Partial | Yes | Moderate concerns |
| Mahundi  2021 | Yes | Yes | Yes | Yes | Yes | No | Yes | Yes | Yes | Minor concerns |
| Manda-Taylor 2022 | Yes | Yes | Yes | Yes | Yes | No | Yes | Yes | Yes | Minor concerns |
| Martin  2017 ^g^ | Yes | Yes | Yes | Yes | Yes | No | Yes | Yes | Yes | Minor concerns |
| Martin  2017 ^h^ | Yes | Yes | Yes | Yes | Yes | No | Yes | Yes | Yes | Minor concerns |
| Mayson  2016 | Yes | Yes | Yes | Yes | Yes | No | Yes | Partial | Yes | Moderate concerns |
| Morrison  2021 | Yes | Yes | Yes | Yes | Yes | No | Yes | Yes | Yes | Minor concerns |
| Morrison  2023 | Yes | Yes | Yes | Yes | Yes | No | Yes | Yes | Yes | Minor concerns |
| Muthuraj  2023 | Yes | Yes | Yes | Yes | Yes | No | Yes | Partial | Yes | Moderate concerns |
| Nahrisah  2019 | Yes | Yes | Yes | No | Yes | No | Yes | Yes | Yes | Moderate concerns |
| Nisar  2014 | Yes | Yes | Yes | Yes | Yes | No | Yes | Yes | Yes | Minor concerns |
| Onyeneho  2016 ^i^ | Yes | Yes | Yes | Yes | Yes | No | Yes | Partial | Yes | Moderate concerns |
| Onyeneho  2016  ^j^ | Yes | No | No | No | Partial | No | Yes | No | Yes | Serious concerns |
| O’Toole  2024 | Yes | Yes | Yes | Yes | Yes | No | Yes | Partial | Yes | Moderate concerns |
| Palmer  2020 | Yes | Yes | Yes | Yes | Yes | No | Yes | Yes | Yes | Minor concerns |
| Pasaribu  2024 | Yes | Yes | Yes | Partial | Partial | Partial | Yes | Partial | Yes | Moderate concerns |
| Riang’a  2020 | Yes | Yes | Yes | Partial | Yes | Partial | Yes | Yes | Yes | Minor concerns |
| Sammartino  2010 | Yes | Yes | Yes | Partial | Yes | No | No | Partial | Yes | Serious concerns |
| Saraswathy  2023 | Yes | Yes | Yes | Partial | Yes | No | Yes | Partial | Yes | Moderate concerns |
| Sedlander  2020 | Yes | Yes | Yes | Yes | Yes | Yes | Yes | Yes | Yes | No or very minor concerns |
| Silubonde  2022 | Yes | Yes | Yes | Yes | Yes | Partial | Yes | Yes | Yes | No or very minor concerns |
| Tancred  2024 | Yes | Yes | Yes | Yes | Yes | Partial | Yes | Yes | Yes | No or very minor concerns |
| Tefera  2023 | Yes | Yes | Yes | Partial | Yes | No | Yes | Partial | Yes | Moderate concerns |
| Tinago  2017 | Yes | Yes | Yes | Yes | Yes | No | Yes | Yes | Yes | Minor concerns |
| Wana  2020 | Yes | Yes | Yes | Partial | Yes | Partial | Yes | Partial | Yes | Moderate concerns |
| Wendt  2018 | Yes | Yes | Yes | Yes | Yes | No | Yes | Yes | Yes | Minor concerns |
| Widyawati  2015 | Yes | Yes | Yes | Partial | Yes | No | Yes | Yes | Yes | Moderate concerns |
| Widyawati  2016 | Yes | Yes | Yes | Partial | Yes | No | Yes | Yes | Yes | Moderate concerns |
| Williams  2020 | Yes | Yes | Yes | Partial | Yes | No | Yes | Yes | Yes | Minor concerns |
| Young  2005 | Yes | Yes | Yes | No | Partial | Yes | Partial | Partial | Yes | Serious concerns |
| Young  2019 | Yes | Yes | Yes | Yes | Yes | No | Yes | Yes | Yes | Minor concerns |

^a^: Implementation fidelity of intravenous ferric carboxymaltose administration for iron-deficiency anaemia in pregnancy: a mixed-methods study nested in a clinical trial in Nigeria

^b^: Acceptability of IV iron treatment for iron deficiency anaemia in pregnancy in Nigeria: a qualitative study with pregnant women, domestic decision-makers, and health care providers

^c^: Acehnese cultural leaders’ perspective on anemia in pregnant women: a qualitative study

^d^: Barriers to health workers in iron deficiency anemia prevention among Indonesian pregnant women

^e^: Husband’s perception on anemia among pregnant women based on cultural perspective: a qualitative study

^f^: Exploring Indonesian mothers’ perspective on anemia during pregnancy: a qualitative study

^g^: Adherence partners are an acceptable behaviour change strategy to support calcium and iron-folic acid supplementation among pregnant women in Ethiopia and Kenya

^h^: Translating formative research findings into a behaviour change strategy to promote antenatal calcium and iron and folic acid supplementation in Western Kenya

^i^: Anaemia is typical of pregnancies: capturing community perception and management of anaemia in pregnancy in Anambra State, Nigeria

^j^: Factors associated with compliance to recommended micronutrients uptake for prevention of anemia during pregnancy in urban, peri-urban, and rural communities in Southeast Nigeria
