# Supplementary material for: Perceptions and experiences of the prevention, testing, and treatment of anaemia in pregnant women: A qualitative evidence synthesis
Source: PLOS Glob Public Health. 2025 Oct 1;5(10):e0005158. doi: 10.1371/journal.pgph.0005158 (PMC12488017; doi:10.1371/journal.pgph.0005158)
Supplement: Attachment — Submitted filename: Response to reviewers.docx [file pgph.0005158.s007.docx]

09 May 2025

Dear PLOS Global Public Health Editorial Team,

We are grateful for the feedback provided by the two reviewers on our manuscript. On Page 2, you will find a table outlining the comments from the reviewers and editorial team, along with our responses. If applicable, we have also included the page and line numbers where changes have been made in the revised manuscript.

Sincerely,

Ebony Verbunt (on behalf of the manuscript’s co-authors)

| **Reviewer feedback** | **Authors’ response** |
| --- | --- |
| **REVIEWER 1** |  |
| I would like to congratulate the authors for this important work. The findings are interesting and represent the issues commonly faced on ground. I have only one small recommendation:  A significant barrier to anemia management is the lack of a POCT that could screen for the cause of anemia. Available POCT (digital hemoglobinometer) in LMICs only screens for the presence of anemia following which the mother is given iron therapy. Available, evidence from India suggests that the prevalence of iron deficiency in mothers with anemia might only be 10-40%. This means that a significant proportion of mothers are getting iron unnecessarily which adds to the GI side effects and also the has the potential to alter gut flora (something that has not been studied much). The existing guidelines in India are also out dated and recommend twice daily iron in anemic mothers when there is enough evidence to show that alternate day iron might work better. I would suggest the authors to include this in the discussion of the paper and recommend future studies on anemia POCTs and oral therapy of iron before focusing on IV iron therapy as there is heavy pharma industry influence in IV iron therapy which in my opinion will fail to improve anemia in LMICs and only add to the cost of anemia management for reasons cited above - only 10-40% have IDA and oral therapy improvement has not been studied extensively. | Thank you very much. We agree with the reviewer that a key challenge of available point-of-care tests is that they do not determine the cause of anaemia (e.g., iron deficiency). This challenge was not discussed by participants in our included studies. We have added the following under ‘Implications for research and policy’ (page 36, lines 642-643).  Our review has also revealed limited evidence on stakeholders’ perceptions and experiences of anaemia testing (e.g., the challenges associated with point-of-care tests in determining the underlying cause of anaemia) and clinical interventions, specifically blood transfusion and intravenous iron.  We agree that there need to be a better understanding of the effectiveness of different doses of supplements. This recommendation is out-of-scope for our paper; however, we have added the following under ‘Implications for research and policy’ (page 36, lines 638-641).  Qualitative and mixed-methods research could explore whether factors influencing women’s adherence to supplements vary by type (e.g., oral iron and folic acid versus multiple micronutrient supplements) and dosage (e.g., daily versus intermittent).  We believe our discussion of intravenous iron is warranted as it specifically addresses its use as a treatment option for iron-deficiency anaemia, rather than anaemia in general. We have added that cost-effectiveness studies have only recently been conducted in low-and middle-income countries (LMICs) (page 35, lines 612-615).  However, intravenous iron as a treatment option for iron-deficiency anaemia is typically not available in LMICs, with clinical trials, implementation science studies, and cost-effective analyses only recently being conducted in Malawi, Nigeria, Bangladesh, and India [100-107]. |
| **REVIEWER 2** |  |
| The ongoing prevalence of anemia during pregnancy, especially in low- and middle-income nations, is a significant worldwide public health concern that is addressed in this publication. The qualitative evidence synthesis has the potential to shape WHO guidelines and prenatal care programs because it is methodologically sound, well-written, and instructive. To improve receptivity and interpretability, a few points need to be clarified,  and slightly revised. | Thank you very much. |
| To support your case of added value, clearly compare your findings with preceding research (such as earlier thematic reviews or individual studies) in the Discussion | Thank you for this suggestion. We note that we have compared our findings to other research about antenatal care (page 34, lines 571-579), supplementation (page 34, lines 586-588), intravenous iron (page 35, lines 595-597), and health system challenges (page 35, lines 604-606).  We have added the following to the manuscript (pages 34-35, lines 605-608).  Overall, our study contributes to the existing body of evidence on factors affecting pregnant women’s engagement with supplementation, complementing a previous study that reviewed barriers and enablers of oral iron and folic acid use across seven countries in Africa and Asia [17].  [17] Siekmans K, Roche M, Kung'u JK, Desrochers RE, De-Regil LM. Barriers and enablers for iron folic acid (IFA) supplementation in pregnant women. Matern Child Nutr. 2018;14 Suppl 5(Suppl 5):e12532. Epub 20171222. doi: 10.1111/mcn.12532. PubMed PMID: 29271115; PubMed Central PMCID: PMCPMC6865983. |
| The section on reflexivity is good. Could be improved by carefully considering how the study participants' professional or cultural proximity may have influenced how the data was interpreted. It reads as general at the moment. | Thank you for raising this observation. We have added an example to the reflexivity statement to demonstrate how our professional proximity may have influenced our interpretation of the data (page 9, lines 201-203).  For example, review authors with a medical background may interpret clinical intervention data more favourably than those with a nutrition background. To address this, we critiqued and challenged our preconceived assumptions through reflexive dialogues, and we supported each other to understand how these assumptions influenced the analysis or interpretation of findings. |
| More specific, feasible recommendations would strengthen the conclusion. What particular interventions (such as task-shifting or mHealth reminders) might be tested in light of the results, for example? | Thank you for raising this important point. We have not suggested potential interventions based on our findings, as we believe that theoretical approaches should be used to identify the most appropriate interventions.  However, we agree that it is important to clearly demonstrate to readers how theoretical approaches can be used to identify appropriate interventions and to provide examples of what these interventions may be. We have therefore added the following information under ‘Implications for research and Policy’ (page 36, lines 614-618).  Future research could map our findings to implementation science frameworks and models, such as the Theoretical Domains Framework (TDF) and the Capability, Opportunity, and Motivation of Behaviour (COM-B) model [110, 111]. This approach would help identify behavioural determinants related to the prevention, testing, and/or treatment of anaemia in pregnant women. The Behaviour Change Wheel (BCW), an intervention development tool, could then be used to select appropriate intervention types – such as training, environmental restructuring and enablement – that specifically targets these determinants and inform the design of potential implementation strategies [111]. |
| Table 2 is arranged neatly. But some conclusions (such 2.11, 2.13, and 3.1) are based on limited evidence. Think about using a footnote or asterisk to visually underline the constraint. | Thank you for this suggestion. In Table 2, under the third column ‘Overall CERQual statement’, we have indicated the confidence level for each finding (very low, low, moderate, or high). To ensure the readers do not overlook this information, we have bolded the text in this column (pages 13-21). We note that this approach is recommended by the GRADE-CERQual methods team and Cochrane Collaboration for reporting Summary of Qualitative Findings and GRADE-CERQual assessments. |
| **EDITOR** |  |
| Please identify your study as “systematic review” in the title of your manuscript | Thank you for your comment. We respectfully note that our study is not a systematic review in the traditional sense. Instead, we have conducted a qualitative evidence synthesis following the Cochrane Effective Practice and Organisation of Care (EPOC) guidance for systematic reviews of qualitative studies [19] (page 6, lines 122-123). Therefore, it would be incorrect to label our study as a “systematic review” in the manuscript title, and we have retained “qualitative evidence synthesis” as the preferred appropriate name of our methodology. We note that this is consistent with current guidance, e.g., by the Cochrane Collaboration for recently published reviews using the same methodology: [Perceptions and experiences of the prevention, identification and management of pre‐eclampsia and eclampsia: a qualitative evidence synthesis](https://www.cochranelibrary.com/cdsr/doi/10.1002/14651858.CD016164/full) and [Perceptions and experiences of the prevention, detection, and management of postpartum haemorrhage: a qualitative evidence synthesis](https://www.cochranelibrary.com/cdsr/doi/10.1002/14651858.CD013795.pub2/full).  [19] Glenton C, Bohren M, Downe S, Paulsen E, Lewin S, on behalf of Effective Practice and Organisation of Care (EPOC). EPOC Qualitative Evidence Syntheses: Protocol and review template v1.3. 2022. doi: <https://doi.org/10.5281/zenodo.5973704>. |
| Please review your reference list to ensure that it is complete and correct. If you have cited papers that have been retracted, please include the rationale for doing so in the manuscript text or remove these references and replace them with relevant current references. Any changes to reference list should be mentioned in the rebuttal letter that accompanies your revised manuscript. If you need to cite a retracted article, indicate the article’s retracted status in the Reference list and also include a citation and full reference for the retraction notice. | We have reviewed our reference list to ensure it is complete and correct. |
