# Supplementary material for: Perceptions and experiences of the prevention, testing, and treatment of anaemia in pregnant women: A qualitative evidence synthesis
Source: PLOS Glob Public Health. 2025 Oct 1;5(10):e0005158. doi: 10.1371/journal.pgph.0005158 (PMC12488017; doi:10.1371/journal.pgph.0005158)
Supplement: Attachment — Submitted filename: Response to reviewer_July.docx [file pgph.0005158.s008.docx]

09 July 2025

Dear PLOS Global Public Health Editorial Team,

We are grateful for the additional feedback provided by the reviewer on our manuscript. On Page 2, you will find a table outlining the comments from the reviewers and editorial team, along with our responses. If applicable, we have also included the page and line numbers where changes have been made in the revised manuscript.

Sincerely,

Ebony Verbunt (on behalf of the manuscript’s co-authors)

| **Reviewer feedback** | **Authors’ response** |
| --- | --- |
| **REVIEWER 2** |  |
| The abstracts and parts of the main document are too lengthy and can be improved with more concise and focused writing. | Thank you for this valuable suggestion. We have carefully reviewed the abstract and main document and have revised the text to improve clarity and conciseness where appropriate (please see track changes). However, we have retained the length of the Methods section to ensure reproducibility, and the Findings section remains unchanged to accurately reflect the scope and depth of included data. |
| Include more concrete policy recommendations or priority actions specific to the WHO guideline component of the health system. | Thank you for this important point. We have added the following under ‘Implications for research and policy’ (pages 36-37, lines 634-641).  Our findings could contribute to the development of future WHO recommendations, particularly around the domains of acceptablity, feasibility, values, and implementation considerations [19]. For example, findings 2.3 to 2.13 may inform the assessment of the acceptability of iron and folic acid supplements for the prevention and treatment of anaemia, whereas findings 2.15, 4.1 and 4.2 might support considerations of the feasibility of intravenous iron treatment.  We also added the following to the conclucion in our abstract and main document (page 3, lines 64-66 and page 37, lines 657-659).  Findings may contribute the development of future WHO recommendations, particularly around the domains of acceptability, feasibility, values, and implementation considerations. |
| **EDITOR** |  |
| Please identify your study as “systematic review” in the title of your manuscript | Thank you for your comment. We respectfully note that our study is not a systematic review in the traditional sense. Instead, we have conducted a qualitative evidence synthesis following the Cochrane Effective Practice and Organisation of Care (EPOC) guidance for systematic reviews of qualitative studies [19] (page 6, lines 122-123). Therefore, it would be incorrect to label our study as a “systematic review” in the manuscript title, and we have retained “qualitative evidence synthesis” as the preferred appropriate name of our methodology. We note that this is consistent with current guidance, e.g., by the Cochrane Collaboration for recently published reviews using the same methodology: [Perceptions and experiences of the prevention, identification and management of pre‐eclampsia and eclampsia: a qualitative evidence synthesis](https://www.cochranelibrary.com/cdsr/doi/10.1002/14651858.CD016164/full) and [Perceptions and experiences of the prevention, detection, and management of postpartum haemorrhage: a qualitative evidence synthesis](https://www.cochranelibrary.com/cdsr/doi/10.1002/14651858.CD013795.pub2/full).  [19] Glenton C, Bohren M, Downe S, Paulsen E, Lewin S, on behalf of Effective Practice and Organisation of Care (EPOC). EPOC Qualitative Evidence Syntheses: Protocol and review template v1.3. 2022. doi: <https://doi.org/10.5281/zenodo.5973704>. |
| Please review your reference list to ensure that it is complete and correct. If you have cited papers that have been retracted, please include the rationale for doing so in the manuscript text or remove these references and replace them with relevant current references. Any changes to reference list should be mentioned in the rebuttal letter that accompanies your revised manuscript. If you need to cite a retracted article, indicate the article’s retracted status in the Reference list and also include a citation and full reference for the retraction notice. | We have reviewed our reference list to ensure it is complete and correct.  The WHO recently published updated data on the prevalence of anaemia in pregnant women. We have accordingly revised the data and cited reference [4].  [4] World Health Organization. Prevalence of anaemia in women of reproductive age (aged 15-49) (%), by pregnancy status [Online]. World Health Organization; 2025 [updated 2025-04-29; cited 2025 26 June]. Available from: <https://www.who.int/data/gho/data/indicators/indicator-details/GHO/prevalence-of-anaemia-in-women-of-reproductive-age-(->). |
