# Supplementary material for: Perceptions and experiences of the prevention, testing, and treatment of anaemia in pregnant women: A qualitative evidence synthesis
Source: PLOS Glob Public Health. 2025 Oct 1;5(10):e0005158. doi: 10.1371/journal.pgph.0005158 (PMC12488017; doi:10.1371/journal.pgph.0005158)
Supplement: Attachment — Submitted filename: Response to reviewer_August.docx [file pgph.0005158.s009.docx]

11 August 2025

Dear PLOS Global Public Health Editorial Team,

We are grateful for the feedback provided by the reviewer on our manuscript. On Page 2, you will find a table outlining the comments from the reviewer, along with our responses. If applicable, we have also included the page and line numbers where changes have been made in the revised manuscript.

Sincerely,

Ebony Verbunt (on behalf of the manuscript’s co-authors)

| **Reviewer feedback** | **Authors’ response** |
| --- | --- |
| **REVIEWER 3** |  |
| This is an excellent synthesis of the available qualitative data on iron interventions for the prevention and treatment of anaemia in pregnancy. | Thank you very much. |
| Can the authors provide a bit more detail in the discussion about the wider context of their work. This could include the types of interventions, their availability and high-level summaries of the use case for these? For an audience who aren’t aware of the nuances in this area of research this would be helpful. | Thank you for your suggestion. We have added further information about treatment options in the ‘Introduction’ section (page 4, lines 85-88).  The World Health Organisation (WHO) recommends pregnant women take daily or intermittent oral iron and folic acid supplements to prevent and treat anaemia [5]. Depending on severity, underlying cause, and individual circumstances, women may be treated for anaemia through other modalities, including multiple micronutrient supplements, blood transfusion, or intravenous iron. For example, in many high-income countries, guidelines recommend intravenous iron for women in the second or third trimester of pregnancy who have moderate to severe iron-deficiency anaemia and either do not respond to, or cannot tolerate, oral iron and folic acid supplements [7-9].  7. American College of Obstetricians and Gynecologists' Committee on Practice Bulletins - Obstetrics. ACOG practice bulletin no. 223: Anemia in pregnancy. Obstet Gynecol. 2021;138(2):55-64. doi: 10.1097/AOG.0000000000004477.  8. National Blood Authority (NBA). Patient Blood Management Guidelines: Module 5 ­­­- Obstetrics and Maternity. Canberra, Australia: NBA, 2015.  9. Pavord S, Daru J, Prasannan N, Robinson S, Stanworth S, Girling J, et al. UK guidelines on the management of iron deficiency in pregnancy. Br J Haematol. 2019;188:819-30. doi: doi.org/10.1111/bjh.16221. |
| Can the authors also provide geographic context for the included studies? I think this is mentioned in the narrative in the section on description of studies, but it would also be helpful to understand if the themes and review findings were across all regions, or in specific areas. | Thank you for your comment. The geographical context of the included studies is provided under the ‘Description of papers’ section (pages 10-11, lines 224-232) and summarised in Table 1, which outlines the characteristics of the included studies.  Additionally, geographical context is one of the elements considered in our GRADE-CERQual assessment of the relevance of each review finding, as shown in Table 2 and detailed in S3 Appendix. We also considered other contextual elements when assessing relevance, including the perspective (e.g., pregnant women, partner/family members, or health workers).  Lastly, under the ‘Confidence in the review findings’ section (pages 12-13, lines 265-266), we note that most studies were conducted in the regions of Africa and South-East Asia (57/64), and in low-income and lower-middle income countries (54/64). |
| There are some areas of repetition (the data described in table 2 are repeated in the narrative). It would be helpful if other information is provided to make clear if such repetition is needed, or actually if additional details are provided that would be preferrable. The authors could consider condensing, however, will need to make a judgement if important details are then lost. | Thank you for your comment. As outlined in the Methods section (page 6, lines 124-125), we followed the Cochrane Effective Practice and Organisation of Care (EPOC) guidance for qualitative evidence syntheses [18].  In line with this guidance, we included the following sub-should headings and content in our Results section:  **Description of papers**   - Characteristics related to our inclusion criteria, as well as other systematically gathered characteristics   **Methodological limitations of papers**   - A description of the methodological limitations of the included studies - A table presenting individual assessments for each study (see S5 Appendix)   **Confidence in the review findings**   - An explanation of our assessment of confidence in the review findings   **Themes and findings from the qualitative evidence synthesis**   - A table summarising our qualitative findings (Table 2), including: - A summary of each review finding - References to contributing studies - The overall GRADE-CERQual assessment of confidence in each finding - An explanation of each GRADE-CERQual assessment - An evidence profile (S3 Appendix), which provides the same information as Table 2, along with the underlying judgements for each GRADE-CERQual component - A detailed description of our qualitative findings, with each finding presented separately, as recommended   [18] Glenton C, Bohren MA, Downe S, Paulsen EJ, Lewin S. Cochrane qualitative evidence synthesis: Protocol and review template. Version 1.4b. Cochrane Person Centred Care, Health Systems and Public Health and Cochrane Norway. 2023. |
| The discussion is short, and provides little context, so could be expanded for the non exert reader, word count permitting. | Thank you for your comment. We believe the Discussion section is of adequate length (1150 words).  We have summarised our review findings and contextualised them within the existing research. For example, we discuss how our finding – that women often normalise symptoms of anaemia and seek treatment only when symptoms become severe – may be explained by Downe and colleagues [92], who found that across diverse settings and contexts, women commonly viewed pregnancy as a normal and healthy state of being (page 34, lines 571-581). We have also contextualised our other findings, including those related to supplements, intravenous iron, and structural health-system deficiencies.  In addition to contextualising our review findings within the broader literature, we have outlined their implications for research and policy (page 36, lines 613-633), as well as the overall strengths and limitations of our study (pages 36-37, lines 635-644). |
